# Supplementary material for: Mechanical properties of discarded shield residue improved by calcium carbide slag and fly ash as subgrade filling
Source: PLoS One. 2024 Dec 3;19(12):e0314597. doi: 10.1371/journal.pone.0314597 (PMC11614222; doi:10.1371/journal.pone.0314597)
Supplement: Attachment — Submitted filename: Comments for PONE-D-24-34216.pdf [file pone.0314597.s002.pdf]

PONE-D-24-34216:

Comments and Suggestions

This manuscript aims to enhance the shield residue with IBPs (i.e. CCS and FA) and utilize it for subgrade filling during the construction of Jihua Road, in order to achieve resource utilization and cost savings. The study presents several techniques to investigate the properties of the samples; the results were also explained and discussed. However, the quality of the manuscript should be further enhanced, particularly the effects of QL substitution by CCS or increasing FA on their properties. The clarifying and potential points are as follows:

Major comments:

Affiliation

- The superscript of afflliliation for all authors should be corrected.

Introduction

1) In litterature reviews with CCR and FA for stabilization soil, plese consider the related recently publications such as :

- Environmentally friendly binders from calcium carbide residue and silica fume and feasibility for soft clay stabilization, https://doi.org/10.1016/j.cscm.2024.e03117
- Evaluation of calcium carbide residue and fly ash as sustainable binders for environmentally friendly loess soil stabilization, https://www.nature.com/articles/s41598-024-51326-x,
- Enhancing durability of concrete mixtures with supplementary cementitious materials: A study on organic acid corrosion and physical abrasion in pig farm environments. https://doi.org/10.1016/j.cscm.2023.e02731
- Acidic corrosion-abrasion resistance of concrete containing fly ash and silica fume for use as concrete floors in pig farm, https://www.sciencedirect.com/science/article/pii/S2214509522001425?via%3Dihub

Materials and methods

2) In XRD patterns, the scale of intensity (Y axis) should be provided and also it should be disscus on the level of crystallity or amprphous phases for all raw materials.
3) There appears to be an inconsistency in using CCS in this study, as the specimen's abbreviation is CCR3FA.
4) Please provide the reason or evidence to support the appropriated experimental misproportions.

Results and discussion

5) Please make sure to thoroughly review and correct the data presented in Table 4. Additionally, kindly provide an explanation and analysis of the results depicted in the table, which indicate variations in moisture content due to the effects of QL substituted by CCS or increasing FA.

6) Please cafully check and correct the data in table 4. Also, please explian and discuss the results in this table which is observed that the moiture content is varied based on the effects of of QL substituted by CCS or increasing of FA.
7) In the CBR test, the results of all specimens should be presented, and the effect of QL substituted by CCS or increasing FA should be discussed.
8) In the UCS test, “After 28 days, the strength growth of the QL-FA stabilized soil slowed down, while that of the CCS-FA stabilized soil continued to progress at a fast pace.” Please explain the reasons for or provide results to support the mechanisms of CCS-FA stabilized soil.
9) Please provide and further discuss the significant results obtained from equation 1, table 5, and Fig. 6.
10) In the moisture content and pH test, please explain the reason to support that those values of CCR are larger than QL specimens.
11) In the water stability test, “This phenomenon could own to the finer particle size and larger specific surface area of CCS, which could lead to a denser microstructure of the stabilized soil.” Please provide the supported results, such as particle size and morphology of QL, CCS, and FA as investigated by SEM measurement.
12) In the Dry-wet cycle test, please explain why the QL3FA4 and CCR3FA4 specimens were selected for testing over other appropriate specimens.
13) In the Dry-wet cycle test, Please provide the results of all specimens in the Dry-wet cycle test and discuss the effects of QL substitution by CCS or increasing FA on this test while enhancing shield residue.
14) In the Dry-wet cycle test, “In comparison with the CCS-FA stabilized soil, the UCS of QL-FA stabilized soil decreased significantly, which is consistent with the apparent state, mass loss, and height changes of the specimens. Hence, it can be concluded that the durability of CCS-FA stabilized soil is superior to QL-FA stabilized soil”. Please provide the results of all speciements and carfully dissussion on the effects of QL substitution by CCS or increasing FA.
15) In the Dry-wet cycle test, “In general, the improvement is mainly due to the finer particle size and larger specific surface area of CCS. The CCS plays a good filling role and makes a denser microstructure of the stabilized soil, leading to greater erosion resistance and chemical stability” I The results of this study have not been supported; please provide the particle size and morphology of QL, CCS, and FA as investigated by SEM measurement.

Conclusions

1) Please summarize the impact of QL substitution by CCS or increasing FA on this study.
2) What are the future studies and proposed applications of shield residual based on their properties?
