# Supplementary material for: Mechanical properties of discarded shield residue improved by calcium carbide slag and fly ash as subgrade filling
Source: PLoS One. 2024 Dec 3;19(12):e0314597. doi: 10.1371/journal.pone.0314597 (PMC11614222; doi:10.1371/journal.pone.0314597)
Supplement: Attachment — Submitted filename: Response to the comments of Editor and Reviewers.doc [file pone.0314597.s003.doc]

**Response to the comments of Editor and Reviewers**

**Manuscript ID:** PONE-D-24-34216

**Title: Mechanical properties of discarded shield residue improved by calcium carbide slag and fly ash**

**Authors: Shoujie Wang, Jianwen Ding, Qingying Guo, Ning Jiao, Chenhao Li**

Thank you very much for your nice suggestions and comments on our manuscript. There is no doubt that these suggestions and comments are valuable and very helpful for revising and improving our manuscript.

We have revised our manuscript carefully and accordingly with regard to all the comments and the suggestions, and the amendments are highlighted in blue in the revised manuscript. The responses to the comments of reviewers are detailed below, in which the paragraphs in **normal fonts** are the comments and the authors’ responses are written in **bold fonts**.

**Response to Editor’s Comments**

1. Please ensure that your manuscript meets PLOS ONE's style requirements, including those for file naming. The PLOS ONE style templates can be found at

https://journals.plos.org/plosone/s/file?id=wjVg/PLOSOne_formatting_sample_main_body.pdf and <https://journals.plos.org/plosone/s/file?id=ba62/PLOSOne_formatting_sample_title_authors_affiliations.pdf>

**Answer:**

**Thank you for your guidance on ensuring that our manuscript adheres to the style requirements of PLOS ONE. We have carefully reviewed and updated our manuscript as requested. Thank you again for your feedback and for providing the resources to assist us in this process.**

2. Please note that funding information should not appear in any section or other areas of your manuscript. We will only publish funding information present in the Funding Statement section of the online submission form. Please remove any funding-related text from the manuscript.

**Answer:**

**Thank you for your instruction regarding the inclusion of funding information in our manuscript. In response to your request, we have thoroughly reviewed our manuscript and have removed all funding-related text from the manuscript.**

3. Thank you for stating the following financial disclosure: “This study is partially supported by the National Natural Science Foundation of China (Grant No. 52378330 and Grant No. 51978159).” Please state what role the funders took in the study. If the funders had no role, please state: "The funders had no role in study design, data collection and analysis, decision to publish, or preparation of the manuscript." If this statement is not correct you must amend it as needed. Please include this amended Role of Funder statement in your cover letter; we will change the online submission form on your behalf.

**Answer:**

**Thank you for your suggestion. In accordance with your instructions, we have included the following statement in our cover letter to ensure the funders' role:**

**"The funders had no role in study design, data collection and analysis, decision to publish, or preparation of the manuscript."**

**Thank you again for your assistance with this matter.**

4. We note that your Data Availability Statement is currently as follows: “All relevant data are within the manuscript and in Supporting Information files.” Please confirm at this time whether or not your submission contains all raw data required to replicate the results of your study. Authors must share the “minimal data set” for their submission.

**Answer:**

**Thank you for pointing out. In response to your comment, we have compiled the relevant data into an Excel file, and thank you again for your assistance with this matter.**

5. Please amend either the title on the online submission form (via Edit Submission) or the title in the manuscript so that they are identical.

**Answer:**

**Thank you for your observation regarding the consistency of the title in our manuscript and the online submission form. Upon receiving your feedback, we have reviewed the title in the manuscript to match the title on the online submission form. We appreciate your attention to detail and thank you for your assistance.**

**Response to Reviewer #1’s Comments**

This manuscript aims to enhance the shield residue with IBPs (i.e. CCS and FA) and utilize it for subgrade filling during the construction of Jihua Road, in order to achieve resource utilization and cost savings. The study presents several techniques to investigate the properties of the samples; the results were also explained and discussed. However, the quality of the manuscript should be further enhanced, particularly the effects of QL substitution by CCS or increasing FA on their properties. The clarifying and potential points are as follows:

**Affiliation**

1. The superscript of affliliation for all authors should be corrected.

**Answer:**

**Thank you for your attention to detail and for pointing out the need for correction in our manuscript. We apologize for the oversight regarding the superscript of affiliation for our authors. Upon receiving your feedback, we have carefully reviewed and corrected the superscript affiliations for all authors to ensure accuracy and consistency throughout the paper.**

**Introduction**

2. In litterature reviews with CCS and FA for stabilization soil, please consider the related recently publications such as:

- Environmentally friendly binders from calcium carbide residue and silica fume and feasibility for soft clay stabilization, https://doi.org/10.1016/j.cscm.2024.e03117

- Evaluation of calcium carbide residue and fly ash as sustainable binders for environmentally friendly loess soil stabilization,

https://www.nature.com/articles/s41598-024-51326-x,

- Enhancing durability of concrete mixtures with supplementary cementitious materials: A study on organic acid corrosion and physical abrasion in pig farm environments. https://doi.org/10.1016/j.cscm.2023.e02731

- Acidic corrosion-abrasion resistance of concrete containing fly ash and silica fume for use as concrete floors in pig farm,

https://www.sciencedirect.com/science/article/pii/S2214509522001425?via%3Dihub

**Answer:**

**Thank you for your suggestion to include more recent high-quality publications in our paper. We have added these in the appropriate location. This suggestion makes the structure of the article more complete. These references are highly valuable, and we have discussed and referenced them in the introduction section. We believe that these revisions have significantly enhanced the depth and breadth of our paper (please refer to reference [13], [14], [18], and [22]).**

**Materials and methods**

3. In XRD patterns, the scale of intensity (Y axis) should be provided and also it should be discus on the level of crystallity or amprphous phases for all raw materials.

**Answer:**

**Thank you for your insightful comments and suggestions. We appreciate your attention to the details of our X-ray diffraction (XRD) analysis. In response to your recommendation, we have now included the scale of intensity on the Y-axis in the XRD patterns presented in our manuscript. This addition will allow readers to more accurately interpret the data and compare the relative intensities of the peaks. Furthermore, we have added our discussion to include an analysis of crystallinity and amorphous phases for all raw materials. (please see lines 68-72)**

**The additions are as follows:**

**Based on the XRD patterns shown in Fig.1, the primary crystalline material in the shield residue was calcite (CaCO3) and quartz (SiO2). And the predominant crystalline material in both QL and CCS was portlandite (Ca(OH)2). As for FA, the broad hump suggested that FA is mainly comprised of amorphous phases with some peaks of Chiastolite (Al2(SiO2)O), quartz, ferric oxide, and lime (please refer to Lines 80-85).**

4. There appears to be an inconsistency in using CCS in this study, as the specimen's abbreviation is CCS3FA.

**Answer:**

**Thank you for pointing this out. The dosage of CCS and QL in this study is consistent. Upon your suggestion, we have confirmed their consistency throughout the experiments. Sorry for causing trouble, we have now corrected the specimen abbreviations from CCS3FA to CCSFA and from QL3FA to QLFA to accurately reflect the content of the material in the paper.**

5. Please provide the reason or evidence to support the appropriated experimental misproportions.

**Answer:**

**Thank you for your inquiry regarding the selection of experimental proportions in our study. In our research, we have carefully considered the selection of our mix proportions before the experiment. The proportions of FA used in our experiments were determined based on preliminary tests and the requirements for subgrade materials. During these pre-experiments, we found that a 2% FA content did not meet the necessary strength and durability for subgrade materials. It can be seen from Fig. 2 and Fig. 4, that the CBR values and UCS of all stabilized soil surpassed the requirement specifications for the design of highway subgrades. Additionally, in line with economic considerations, we have chosen 3% lime with 3%, 4%, and 6% for the experiment. The corresponding mix proportion of 3% CCS with varying percentages of FA (3%, 4%, and 6%) was also theoretically chosen for the experiment.**

**Results and discussion**

6. Please make sure to thoroughly review and correct the data presented in Table 4. Additionally, kindly provide an explanation and analysis of the results depicted in the table, which indicate variations in moisture content due to the effects of QL substituted by CCS or increasing FA.

**Answer:**

**Thanks to the Reviewer’s comment. We appreciate your request for a thorough examination of the data and an in-depth analysis of the results. In response to your feedback, we have carefully reviewed and made the necessary corrections to the data in Table 4 to ensure its accuracy. We have also added and corrected the analysis of the variations of optimum moisture content and maximum dry density of stabilized soil in Section 3.1 (please refer to Lines 107-119).**

**The revised content is as follows:**

**It could be observed that after adding improved materials to the shield residue, the optimum moisture content of the stabilized soil increased, while the maximum dry density decreased somewhat. This might be attributed to the lower density of the improved material compared to shield residues, as well as some flocculent cementation products that result in a certain volume expansion, thereby reducing soil compactness and density [34-35]. Conversely, the incorporation of FA could fill soil pores and the greater specific surface area of FA could facilitate the formation of bound water as water molecules infiltrate the micropores [15]. As a result, the optimal water content of stabilized soil increased with the increase of FA [20, 36]. Besides, FA was prone to pozzolanic reactions with QL and CCS to generate cementitious materials. This reaction consumed a large amount of water, implying additional water needs to be added in order to obtain the stabilized soil with optimal water content. Consequently, as the FA content increased, the optimum moisture content of the stabilized soil also increased [16]**

7. Please carefully check and correct the data in table 4. Also, please explian and discuss the results in this table which is observed that the moiture content is varied based on the effects of of QL substituted by CCS or increasing of FA.

**Answer:**

**Thank you for your reminder. We appreciate your request for a thorough examination of the data and an in-depth analysis of the results. In response to your feedback, we have carefully checked and corrected the date in Table4 to ensure its accuracy. And we have added the analysis of the variations of optimum moisture content and maximum dry density of stabilized soil in Section 3.1 (please refer to Lines 107-119). The revised content can refer to the response provided in question 7.**

8. In the CBR test, the results of all specimens should be presented, and the effect of QL substituted by CCS or increasing FA should be discussed.

**Answer:**

**Thank you for your valuable suggestion regarding the presentation of CBR test results. We have taken your guidance seriously and have now included the CBR value and swelling rate of the raw shield residue, which were 8.1% and 3.0%, respectively, in Section 3.2 of our manuscript (please refer to lines 126-127).**

**Furthermore, in line with your recommendation, we have checked and corrected the Fig. 2 and Fig. 3 to ensure that all CBR test results for the stabilized soil are presented. Additionally, we have expanded and corrected Section 3.2 to include a more detailed discussion on the effects when QL is substituted by CCS and with the increasing addition of FA .**

**The effects on QL are substituted by CCS and with the increasing addition of FA can be concluded as follows (please refer to lines 135-142 and lines 148-152):**

**These CBR values of CCS-FA stabilized soil were slightly lower than those of the QL-FA stabilized soil at the same FA content. The incorporation of FA could also enhance the bearing capacity of CCS-FA stabilized soil and QL-FA stabilized soil. Furthermore, the swelling rate of the QL-FA stabilized was slightly lower compared to CCS-FA stabilized soil, but the difference between the two is small, which indicated that the swelling trend of both stabilized soils was not significantly different.**

**The impact of curing time on the CBR values was slightly lower at the initial stage for the CCS-FA stabilized soil than that of the QL-FA stabilized soil. However, the CBR values exceeded those of the QL-FA stabilized soil slightly at 28 days. In addition, the swelling rate of both stabilized soils substantially decreased, reducing to within 0.1% after 7 days of standard curing.**

9. In the UCS test, “After 28 days, the strength growth of the QL-FA stabilized soil slowed down, while that of the CCS-FA stabilized soil continued to progress at a fast pace.” Please explain the reasons for or provide results to support the mechanisms of CCS-FA stabilized soil.

**Answer:**

**Thank you for pointing this out. We have analyzed the strength curves presented in Figure 4, which reveal that while the QL-FA stabilized soil exhibited a rapid strength increase in the first 28 days, its rate of strength gain slowed down subsequently. In contrast, the CCS-FA stabilized soil maintained a more sustained pace of strength development beyond the initial 28 days.**

**To provide a clearer understanding, we have calculated the average daily strength growth rates for both types of stabilized soils. For the QL-FA stabilized soil, the rate was 16.0 kPa per day during the first 28 days and slowed to 1.9 kPa per day from days 28 to 60. On the other hand, the CCS-FA stabilized soil showed a growth rate of 11.4 kPa per day in the first 28 days, which decreased but remained higher at 6.0 kPa per day from days 28 to 60. This phenomenon indicates that the strength growth rate of CCS-FA stabilized soil is greater than that of QL-FA stabilized solidified soil after initial 28 days.**

**Sorry for causing trouble, we have addad the reasons of the phenomenon in Section 3.3 (please refer to Lines 181-184).**

10. Please provide and further discuss the significant results obtained from equation 1, table 5, and Fig. 6.

**Answer:**

**Thank you for your suggestion to further discuss the significant results obtained from Equation 1, Table 5, and Figure 6. In response to your feedback, we have incorporated a detailed discussion of the results derived from Equation 1, the data presented in Table 5, and the observations from Figure 6 within Section 3.3 of our manuscript (please refer to Lines 214-219).**

**The revised content is as follows:**

**The hyperbolic function could effectively fit the characteristic curves of UCS as it varies with the curing time. Analysis of the regression parameters revealed that the variation range for parameter p1 is relatively small and was not significantly affected by the type or content of the improved materials, remaining between 1.94 and 2.14. In contrast, variation in material content had a more significant impact on parameter p2, which ranges from 2.73 to 3.50.**

11. In the moisture content and pH test, please explain the reason to support that those values of CCS are larger than QL specimens.

**Answer:**

**Thank you for your inquiry regarding the moisture content and pH values in the CCS-FA stabilized soil compared to the QL-FA stabilized soil. The variations in moisture content and pH can be attributed to the extent of hydration reactions and pozzolanic reactions that occur in lime-based and supplementary cementitious materials, respectively. The hydration reaction and pozzolanic reactions, which is more pronounced in the QL-FA stabilized soil, consumes moisture and hydroxide ions from the soil, leading to a decrease in moisture and hydroxide ions content. Compared with the QL-FA, the CCS-FA stabilized soil exhibit a lower degree of reaction. This phenomenon is also verified by the fact that the UCS of the QL-FA stabilized soil is slightly greater than that of the CCS-FA stabilized soil. Thank you again for your insightful comments and we hope the explanation satisfies your query.**

12. In the water stability test, “This phenomenon could own to the finer particle size and larger specific surface area of CCS, which could lead to a denser microstructure of the stabilized soil.” Please provide the supported results, such as particle size and morphology of QL, CCS, and FA as investigated by SEM measurement.

**Answer:**

**Thank you for your insightful comment on the analysis of the water stability test. While we have not conducted SEM measurements, we have performed particle size distribution tests that provide valuable insights into the microstructure of the stabilized soil. Our results indicated that the content of CCS particles with a diameter less than 75 µm is 88%, significantly higher than the 53% observed for QL. This suggests that CCS has a greater proportion of finer particles and a larger specific surface area, contributing to a denser microstructure in the stabilized soil.**

**Although the SEM analysis was not part of our initial study, we recognize the importance of investigating the microstructure in depth. Therefore, we plan to conduct further research on the micro characteristics of the stabilized soil, such as SEM, thermo gravimetric analysis.**

**We appreciate your suggestion and thank you again for your valuable feedback.**

13. In the Dry-wet cycle test, please explain why the QL3FA4 and CCS3FA4 specimens were selected for testing over other appropriate specimens.

**Answer:**

**Thanks to the Reviewer’s comment. The choice of QL3FA4 and CCS3FA4 specimens for the dry-wet cycle test was based on a comprehensive evaluation of both the mechanical performance and economic viability of the stabilized soil.**

**Initially, we focused on the unconfined compressive strength (UCS) and California bearing ratio (CBR) values of the stabilized soil, as depicted in Figures 2 and 4. Our analysis revealed that the UCS of QL-FA stabilized soil with 4% and 6% FA incorporation was almost the same, and the CBR value of QL-FA stabilized soil with 6% FA incorporation was only slightly higher than that of stabilized soil with 4% FA. In line with economic considerations, the lower total dosage of 3% QL + 4% FA (QLFA4) was selected for the dry-wet cycle test. Hence, the corresponding mix proportion of 3% CCS + 4% FA (CCSFA4) was also theoretically chosen for the dry-wet cycle test.**

**Thank you again for your valuable feedback. We have now expanded upon these reasons in Section 3.6 of our manuscript, providing a detailed justification for the selection of QLFA4 and CCSFA4 specimens for the dry-wet cycle test (please refer to lines 260-265).**

14. In the Dry-wet cycle test, Please provide the results of all specimens in the Dry-wet cycle test and discuss the effects of QL substitution by CCS or increasing FA on this test while enhancing shield residue.

**Answer:**

**Thank you for your valuable advice, which highlighting the importance of including dry-wet cycle test results for all specimens in our study and provides the direction for our further research.**

**We agree that that more research would be useful to understand the effect of QL substitution by CCS or increasing FA. Regrettably, in our current study, we have only conducted dry-wet cycle tests on the QLFA4 and CCSFA4 specimens. The results, including mass loss rate and height change under dry-wet cycling conditions, were presented in Figure 12. Additionally, the UCS of the specimens under the standard curing conditions and dry-wet cycle were presented in Fig. 13. We have added our reasons for selecting QLFA4 and CCSFA4 specimens for the dry-wet cycle test in Section 3.6 of our manuscript (please refer to lines 260-265). Moving forward, we plan to conduct the dry-wet cycle test on specimens of all mix proportions to further explore the effect. This additional research will provide a more comprehensive understanding of the soil stabilization process and its durability under varying conditions.**

**Thank you again for your insightful comments. We have also added some analysis of the substitution of QL by CCS in the QLFA4 and CCSFA4 specimens within Section 3.6 (please refer to lines 284-286 and lines 302-303).**

15. In the Dry-wet cycle test, “In comparison with the CCS-FA stabilized soil, the UCS of QL-FA stabilized soil decreased significantly, which is consistent with the apparent state, mass loss, and height changes of the specimens. Hence, it can be concluded that the durability of CCS-FA stabilized soil is superior to QL-FA stabilized soil”. Please provide the results of all speciements and carfully dissussion on the effects of QL substitution by CCS or increasing FA.

**Answer:**

**Thank you for your valuable advice, which highlighting the importance of including dry-wet cycle test results for all specimens in our study and provides the direction for our further research.**

**There is no doubt that the addition of dry-wet cycle test to all specimens will help to understand the effect of QL substitution by CCS or increasing FA. Regrettably, in our current study, we only selected the QLFA4 and CCSFA4 specimens for dry-wet cycle test. The variation of mass loss rate and height change of QLFA4 and CCSFA4 specimens under dry-wet cycle were presented in Fig. 12. And the UCS of the specimens under the standard curing conditions and dry-wet cycle were all presented in Fig. 13. Sorry for causing trouble, and we have expanded upon these reasons in Section 3.6 of our manuscript, providing a detailed justification for the selection of QLFA4 and CCSFA4 specimens for the dry-wet cycle test (please refer to lines 260-265). In the future, the dry-wet cycle test will be carried out on specimens of all mix proportions to further explore the effect of QL substitution by CCS and increasing addition of FA**

**Thank you again for your valuable feedback. We have also added some analysis of the substitution of QL by CCS in the QLFA4 and CCSFA4 specimens within Section 3.6 (please refer to lines 284-2860 and lines 302-303).**

16. In the Dry-wet cycle test, “In general, the improvement is mainly due to the finer particle size and larger specific surface area of CCS. The CCS plays a good filling role and makes a denser microstructure of the stabilized soil, leading to greater erosion resistance and chemical stability” I The results of this study have not been supported; please provide the particle size and morphology of QL, CCS, and FA as investigated by SEM measurement.

**Answer:**

**Thank you for your insightful comment on the analysis of the water stability test. While we have not conducted SEM measurements, we have performed particle size distribution tests that provide valuable insights into the microstructure of the stabilized soil. Our results indicated that the content of CCS particles with a diameter less than 75 µm is 88%, significantly higher than the 53% observed for QL. This suggests that CCS has a greater proportion of finer particles and a larger specific surface area, contributing to a denser microstructure in the stabilized soil.**

**Although the SEM analysis was not part of our initial study, we recognize the importance of investigating the microstructure in depth. Therefore, we plan to conduct further research on the micro characteristics of the stabilized soil, such as SEM, thermo gravimetric analysis.**

**We appreciate your suggestion and thank you again for your valuable feedback.**

**Conclusions**

17. Please summarize the impact of QL substitution by CCS or increasing FA on this study.

**Answer:**

**Thank you for your suggestion to summarize the impact on usage CCS instead of QL and increasing the content of FA in our study. Substituting CCS for QL initially resulted in a slight decrease in both the CBR values and UCS of the specimens. However, the long-term strength of the CCS-FA stabilized soil became comparable to that of the QL-FA stabilized soil. Importantly, using CCS instead of QL enhanced the water stability and durability of the stabilized soil. The CCS could effectively substitute QL to enhance the mechanical properties of shield residue as subgrade filling.**

**Thank you again for your valuable feedback. We have added the summarization in the Section 4 of our manuscript (please refer to lines 331-335).**

18. What are the future studies and proposed applications of shield residual based on their properties?

**Answer:**

**Thanks to the Reviewer’s comment. In response to your suggestion, we have expanded our future research plan, which is now detailed in Section 4 of our manuscript (please refer to lines 336-342). Future investigations will focus on the following aspects for exploration. First, the dry-wet cycle test will be carried out on specimens of all mix proportions to further explore the effect of QL substitution by CCS and the increasing addition of FA. Second, performing microscopic tests such as scanning electron microscopy and thermal gravimetric analysis to gain a deeper understanding of the soil's microstructure and mechanical mechanism. In addition, the study will investigate the feasibility of using soda residue, flue gas desulfurization gypsum, or a combination of diverse supplementary cement materials in conjunction with CCS as substitutes for lime in subgrade filling.**

**Thank you again for your insightful comments.**

**Response to Reviewer #2’s Comments**

This manuscript comprehensively analyzes the improvement of mechanical properties of shield residue by calcium carbide slag and fly ash, and obtains some reliable results. An innovative idea of recycling engineering waste is proposed, which has certain research value, but there are still some deficiencies. The comments and suggestions are as follows.

1. Sec. 3.3, there is a lack of strength and other mechanical parameters of shield muck samples, it is recommended to be supplemented.

**Answer:**

**Thank you for pointing this out. The initial data of shield residue is indeed very important. The CBR value and swelling rate of the raw shield residue were 8.1% and 3.0%, respectively. And the UCS of the raw shield material specimen was 0.44 MPa. We have added those data of the shield residue in Section 3.2 and Section 3.3, respectively (please refer to lines 126-127, 154-155).**

**Thank you again for your valuable feedback.**

2. Sec.3.3, the reaction trends of QL-FA and CCS-FA are not the same, please explain further.

**Answer:**

**Thank you for your valuable comments regarding the differing reaction trends between QL-FA and CCS-FA. We have analyzed the strength curves presented in Figure 4, which reveal that while the QL-FA stabilized soil exhibited a rapid strength increase in the first 28 days, its rate of strength gain slowed down subsequently. In contrast, the CCS-FA stabilized soil maintained a more sustained pace of strength development beyond the initial 28 days.**

**To provide a clearer understanding, we have calculated the average daily strength growth rates for both types of stabilized soils. For the QL-FA stabilized soil, the rate was 16.0 kPa per day during the first 28 days and slowed to 1.9 kPa per day from days 28 to 60. On the other hand, the CCS-FA stabilized soil showed a growth rate of 11.4 kPa per day in the first 28 days, which decreased but remained higher at 6.0 kPa per day from days 28 to 60. This phenomenon indicates that the strength growth rate of CCS-FA stabilized soil is greater than that of QL-FA stabilized solidified soil after initial 28 days.**

**Additional, we have added some analysis of phenomenon. This phenomenon might be attributed to the more intense pozzolanic and ion exchange reactions occurring in the QL-FA stabilized soil during the initial stage. However, the higher pH value provided the necessary alkaline excitation environment, and the active components of CCS (SiO2 at 4.88% and Al2O3 at 1.94%) were higher than those of QL (SiO2 at 3.26% and Al2O3 at 0.46%), which led to a more repid subsequent strength growth of CCS-FA stabilized soil.**

**Sorry for causing trouble, we have addad the reasons and the analysis of the phenomenon in Section 3.3 (please refer to Lines 181-184 and lines 186-190). Thank you again for your insightful comments.**

3. Sec.3.1, line 117, please explain why the higher the content of FA, the smaller the particle size, or give the specific steps for making samples with different proportions?

**Answer:**

**Thank you for your question regarding the relationship between the content of FA and particle size. We apologize for causing trouble. The main purpose of this sentence (line 117) is to express that FA has a small particle size and a large specific surface area, which has a filling effect on the pores of solidified soil. In response to your feedback, we have corrected the expression and added the analysis of the effect of increasing FA (please refer to line 107-119).**

**The revised content is as follows:**

**It could be observed that after adding improved materials to the shield residue, the optimum moisture content of the stabilized soil increased, while the maximum dry density decreased somewhat. This might be attributed to the lower density of the improved material compared to shield residues, as well as some flocculent cementation products that result in a certain volume expansion, thereby reducing soil compactness and density [34][35]. Conversely, the incorporation of FA could fill soil pores and the greater specific surface area of FA could facilitate the formation of bound water as water molecules infiltrate the micropores [15]. As a result, the optimal water content of stabilized soil increased with the increase of FA [20, 36]. Besides, FA was prone to pozzolanic reactions with QL and CCS to generate cementitious materials. This reaction consumed a large amount of water, implying additional water needs to be added in order to obtain the stabilized soil with optimal water content. Consequently, as the FA content increased, the optimum moisture content of the stabilized soil also increased [16]**

4. Only the results of 3 % CCS and 3 %, 4 %, 6 % FA content are analyzed, are they representative?

**Answer:**

**Thank you for your insightful comment. In our research, we have carefully considered the selection of our mix proportions before the experiment and we think the experimental proportion we selected is representative to a certain extent. The purpose of this study is to investigate the credibility of using CCS instead of QL to achieve the utilization of discarded shield residue and alleviate the shortage of subgrade filling. The lime content can be adjusted within the range of 3% and 9% when treating soil with a high plasticity index.**

**Based on preliminary tests and related literature, we chose the proportion of 3% QL and the varying percentages of FA (3%, 4%, and 6%) for the experiment. It can be seen from Fig. 2 and Fig. 4, that the CBR values and UCS of all stabilized soil surpassed the requirement specifications for the design of highway subgrades. Additionally, in lines with economic considerations, we have chosen 3% lime with 3%, 4%, and 6% for the experiment. The corresponding mix proportion of 3% CCS with varying percentages of FA (3%, 4%, and 6%) was also theoretically chosen for the experiment.**

**Thank you again for your valuable feedback.**

5. In section of Conclusion, It is suggested to explain the limitations of the research, as well as the prospect and improvement direction of the research.

**Answer:**

**Thank you for your suggestion. In response to your comment, we have revised the Section 4 of our manuscript to include the prospect for the experiment (please refer to lines 336-342). Future investigations will focus on the following aspects for exploration. First, the dry-wet cycle test will be carried out on specimens of all mix proportions to further explore the effect of QL substitution by CCS and increasing addition of FA. Then, performing microscopic tests such as scanning electron microscopy and thermal gravimetric analysis to gain a deeper understanding of the soil's microstructure and mechanical mechanism. In addition, the study will investigate the feasibility of using soda residue, flue gas desulfurization gypsum, or a combination of diverse supplementary cement materials in conjunction with CCS as substitutes for lime in subgrade filling.**

**Thank you again for your insightful comments.**
